# Fenofibrate attenuates the adverse effects of radiation on endothelial cells through modulation of ROS-NO signalling and inflammation

**DOI:** 10.1016/j.redox.2025.103994

**Published:** 2025-12-25

**Authors:** Omar Elsaeed, Christine von Törne, Prabal Subedi, Louis Wilhelm, Lukas Duchrow, Ali Bashiri Dezfouli, Gabriele Multhoff, Simone Moertl, Omid Azimzadeh

**Affiliations:** a Section Radiation Biology, Federal Office for Radiation Protection, Munich, Germany; b Metabolomics and Proteomics Core (MPC), Helmholtz Zentrum München - German Research Centre for Environmental Health GmbH, Munich, Germany; c Central Institute for Translational Cancer Research (TranslaTUM), TUM School of Medicine and Health, Technical University of Munich, Munich, Germany; d Department of Radiation Oncology, Campus Klinikum rechts der Isar, TUM School of Medicine and Health, Technical University of Munich, Munich, Germany; e Department of Otolaryngology, Head and Neck Surgery, TUM School of Medicine and Health, Technical University of Munich, Munich, Germany

**Keywords:** Ionizing radiation, Proteomics, Heart, Endothelial cells, PPARα, Fenofibrate, Interferon, Cardiac inflammation, NO, ROS, ISG15, Cardiovascular disease

## Abstract

Exposure to high doses of ionizing radiation has been consistently associated with the development of cardiovascular disease (CVD), particularly in patients undergoing thoracic radiotherapy. Radiation-induced CVD is characterized by alterations in metabolic and inflammatory homeostasis in the heart and vascular system. These changes promote myocardial and vascular pathological remodelling. Over time, such changes contribute to the development of atherosclerosis and heart failure. A key driver of these alterations is inactivation of the transcription factor peroxisome proliferator-activated receptor alpha (PPARα). Activation of PPARα by its agonist, fenofibrate, has shown to reduce the irradiation effects on the cardiac proteome of mice. However, the molecular effects of fenofibrate on individual cell types, including vascular endothelial cells, are unknown.

To assess the potential effects of fenofibrate on irradiated vascular endothelium, we performed label-free proteomic analysis of human coronary artery endothelial cells treated with fenofibrate (10 μM) or DMSO at 2- and 7-days post-irradiation (4 Gy). The alterations in expression level and activity status of crucial proteins and mRNA contributing to the affected pathways were further validated. Fenofibrate effectively restored critical molecular and cellular processes disrupted by irradiation, including cell survival, oxidative stress response, ROS production, PI3K-AKT-eNOS signalling, NO bioavailability, inflammatory responses via interferon signalling and endothelial-to-mesenchymal transition. Taken together, these findings further highlight the involvement of PPARα in modulating the cardiovascular response to radiation exposure. Accordingly, the results suggest that administering fenofibrate may have a benefit in preventing vascular disease after radiation exposure. Preventing or mitigating endothelial injury, and inflammation represents a key strategy to preserve vascular integrity and improving long-term cardiovascular outcomes in cancer survivors following radiotherapy.

## Highlights

• Radiation-induced CVD is associated with persistent metabolic and inflammatory dysregulation, primarily due to PPARα inactivation.
• Fenofibrate restores the adverse effects of irradiation on endothelial cells structure and function.
• Radioprotective effects of fenofibrate is mediated through modulation of ROS–NO signalling and inflammatory pathways.

## Graphical abstract

**Figure:**
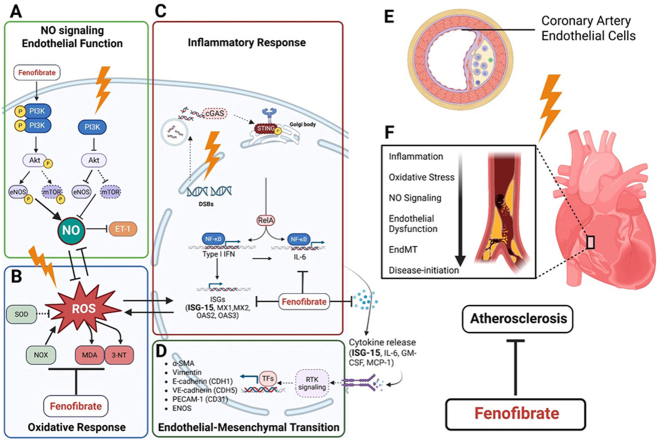

## 1 Introduction

Exposure to high doses of ionizing radiation has been associated with the development of cardiovascular disease (CVD), particularly in patients undergoing thoracic radiotherapy [[1], [2], [3], [4]]. Patients treated with radiotherapy for left-sided breast cancer have a significant increase in cardiovascular mortality compared with those with right-sided cancer [1].

Radiation-induced CVD is characterized by long-term, late-onset adverse effects on the structure and function of the myocardium and vasculature, contributing to progressive cardiac injury. Vascular endothelial cells are among the primary targets of radiation exposure and play a central role in the development of radiation-induced CVD [5]. Irradiation can trigger endothelial dysfunction, characterized by impaired nitric oxide (NO) production, increased oxidative stress, and a pro-inflammatory state [6]. These events contribute to pathological vascular remodelling, including structural changes, intimal thickening and functional impairments in vasodilation, ultimately promoting the development of atherosclerosis hypertension and coronary artery disease [7]. Emerging evidence indicates that radiation-induced CVD is associated with persistent dysregulation of metabolism and inflammation mainly due to inactivation of their key regulator, peroxisome proliferator-activated receptor alpha (PPARα) [[8], [9], [10]]. However, the mechanisms by which disruption of PPARα-regulated pathways contributes to vascular damage in the irradiated heart are not yet fully elucidated.

PPARα, a nuclear receptor that regulates cardiac metabolic processes and inflammatory responses, contributes to physiology and pathology of heart and vasculature [11,12]. PPARα transcriptional activity depends on endogenous (e.g., fatty acids) and exogenous ligands (e.g., fibrates) [13], making synthetic PPARα agonists promising therapeutic agents in cardiac disease [14]. Beyond lipid-lowering, fibrates also reduce vascular inflammation and slow premature coronary atherosclerosis [15,16]. Our previous work demonstrated that activation of PPARα with fenofibrate effectively mitigated irradiation-induced alterations in the cardiac proteome of mice [17]. However, the impact of fenofibrate on the vascular endothelium following radiation exposure remains unclear.

Therefore, this study aimed to evaluate the effects of fenofibrate on endothelial cells exposed to irradiation. To achieve this, we compared the proteome of primary human coronary artery endothelial cells (HCAEC) treated with fenofibrate (10 μM) or DMSO at 2- and 7-days (d) post-irradiation (4 Gy). The key-findings from the mass spectrometry (MS)-based proteome profiling were further validated on the mRNA and protein levels. Additionally, the effect of fenofibrate on radiation-induced cell death, as well as on the production of reactive oxygen species (ROS) and NO and inflammatory responses was analyzed. The results presented in this study suggest that activation of the PPARα signalling pathway has a positive impact on the outcome of radiation-induced vasculature injury.

## 2 Results

### 2.1 Low dose concentration of fenofibrate did not affect HCAECs cell survival

To determine the non-toxic concentration of fenofibrate for HCAECs, the cell toxicity was examined 2d and 7d after treatment with different concentration of fenofibrate using Presto Blue high sensitivity (PB-HS) assay. The results show no significant changes in HCAECs cell survival, 2d after treatment with fenofibrate in a concentration up to 50 μM. However, the viability of cells significantly declined almost 15 %, after treatment with 100 μM fenofibrate (Supplementary Fig. 1A). The survival fraction of the cells was reduced 7d after treatment with 25, 50 and 100 μM of fenofibrate significantly (Supplementary Fig. 1B). Therefore, all further experiments were performed using 10 μM fenofibrate.

### 2.2 Fenofibrate attenuated apoptosis and improved cell viability in irradiated cells

**Fig. 1:**
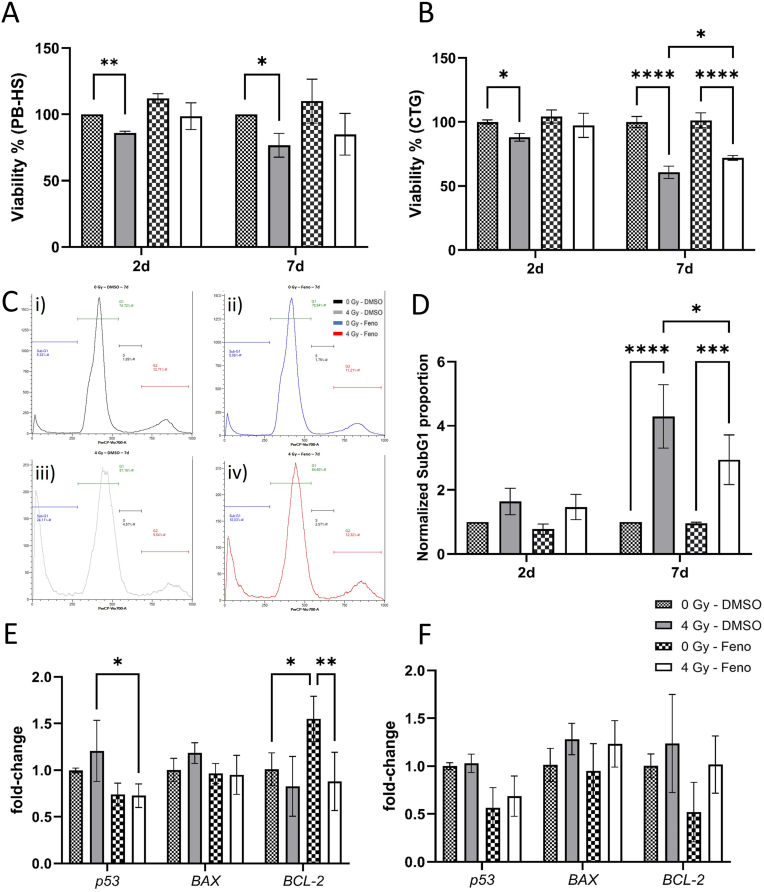
**The effect of fenofibrate on HCAECs viability and apoptosis**. The viability of HCAECs treated with fenofibrate (Feno) was measured using Presto Blue high sensitivity assay (PB-HS) (A) and Cell Titer Glo (CTG) assay (B) 2d and 7d after irradiation. The cell cycle distribution was measured by PI staining and flow cytometry (C). An example of the measurement for the 7d time point is shown for the 0 Gy DMSO (i), 0 Gy fenofibrate (ii), 4 Gy DMSO (iii), and 4 Gy fenofibrate (iv). The subG1 proportion of the cells was quantified as an assessment for apoptosis and normalized for the cell counts (per 10^6^ cells) (D). The apoptotic markers were measured using qPCR, 2d (E) and 7d (F) after irradiation. The error bars represent the standard deviation (±SD). For the Presto Blue assay, multiple unpaired t-tests with a 1 % FDR, Two-stage step-up (Benjamini, Krieger, and Yekutieli) correction was conducted. For the CTG, qPCR and SubG1 analysis, a Two-way ANOVA, Tukey's multiple comparisons test was performed (∗*p* ≤ 0.05; ∗∗p ≤ 0.01; ∗∗∗*p* ≤ 0.001; ∗∗∗∗*p* ≤ 0.0001; n = 3).

To evaluate whether fenofibrate treatment protects the HCAECs from radiation-induced cell death, the cell viability in response to radiation exposure combined with fenofibrate was examined with PB-HS assay 2d and 7d post irradiation. Irradiation significantly reduced the viability of HCAEC at both time points. However, pre-treatment with fenofibrate attenuated this decline, resulting in no statistically significant difference compared to non-irradiated, fenofibrate-treated controls (Fig. 1A).

Additionally, cellular ATP as a direct assessment of metabolic activity and cellular viability, was measured using CellTiter-Glo (CTG) assay. Irradiation has shown to significantly reduce the viability of HCAEC at both time points, whereas fenofibrate treated cells showed no significant reduction in viability 2d after irradiation, indicating a supportive effect of fenofibrate. At 7d timepoint, the irradiated cells treated with fenofibrate showed higher viability compared to 4 Gy–DMSO cells (Fig. 1B).

To assess the effects of fenofibrate treatment on the induction of apoptosis, late-stage apoptosis was assessed using a Sub-G1 assay. Furthermore, the differential expression levels of apoptosis regulators such as *p53*, *BAX* and *BCL2* were analyzed using qPCR. Primers and primer sequences used are listed in Supplementary Table S1. The analysis showed that fenofibrate reduced the proportion of apoptotic Sub-G1 cells 7d after irradiation in comparison to the irradiated DMSO-treated cells (Fig. 1C and D). Fenofibrate treatment decreased significantly the *p53* induction 2d after irradiation. Fenofibrate increased the expression of the anti-apoptotic gene *BCL-2* but only in non-irradiated cells. At 7d time point, fenofibrate did not affect radiation-induced expression of apoptosis markers (Fig. 1E and F).

### 2.3 Fenofibrate increased PPARα phosphorylation and expression of its target genes

**Fig. 2:**
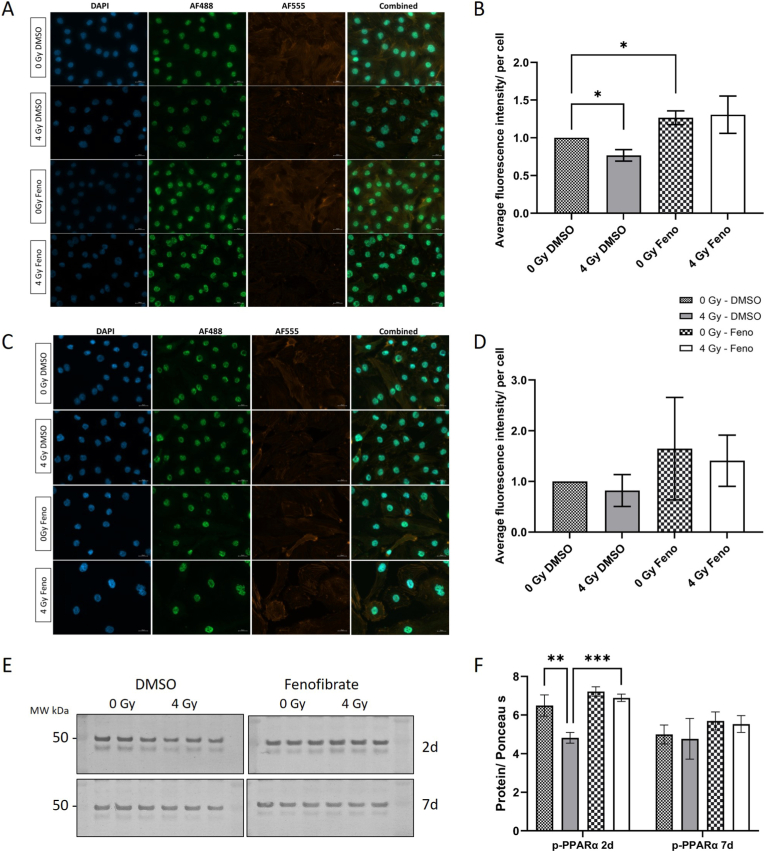
**Changes in the PPARα phosphorylation in irradiated HCAECs with fenofibrate treatment**. PPARα phosphorylation was measured using fluorescence microscopy and western blots. For microscopy, the nucleus was stained with DAPI, the phospho-Ser12 PPARα was visualized using the AF488 channel and the actin-filaments with the AF555 channel at the 2d (A and B) and 7d (C and D) after irradiation with and without fenofibrate (Feno). Images were acquired using identical exposure times and sensitivity settings for all treatments at each time point. Fluorescence signals from the AF488 channel were quantified and normalized to the number of cells per frame (B–D). Immunoblotting of phospho-Ser12 PPARα and quantification is shown in (E) and (F). The error bars represent the standard deviation (±SD). For microscopy, multiple unpaired t-tests with a 1 % FDR, Two-stage step-up (Benjamini, Krieger, and Yekutieli) correction was conducted. For immunoblotting, a Two-way ANOVA, with Tukey's multiple comparisons test was conducted (∗*p* ≤ 0.05; ∗∗*p* ≤ 0.01; ∗∗∗*p* ≤ 0.001; ∗∗∗∗*p* ≤ 0.0001; n = 3).

Since the activity of PPARα is regulated by phosphorylation [18,19], we analyzed the effects of radiation and fenofibrate treatment on the phosphorylated PPARα at Serine (Ser12) using fluorescence microscopy and immunoblotting. Both methods revealed a decrease in (Ser12) phosphorylation, 2d after 4 Gy exposure, suggesting reduced PPARα activity after irradiation. Microscopy data showed that fenofibrate treatment significantly increased PPARα phosphorylation in HCAECs (Fig. 2A and B), in absence of irradiation. A similar pattern of (Ser12) phosphorylation was observed at the 7 d time point; however, greater variability among the quantified cells prevented statistical significance (Fig. 2C and D). The results of immunoblotting confirmed that irradiation significantly reduced phosphorylated PPARα at 2d, and fenofibrate treatment restored phosphorylation at this early time point. By 7d, the slight increase in phosphorylated PPARα in fenofibrate-treated cells was not statistically significant (Fig. 2E and F and Supplementary Fig. 2A–D).

To validate whether enhanced phosphorylated PPARα alters its activity, we analyzed the effect of fenofibrate on the mRNA expression of some of PPARα transcriptional targets [20,21] related to metabolism (Glucose transporter 1 - *Glut1* and pyruvate dehydrogenase kinase 1 - *PDK-1*), inflammation (Nuclear Factor NF-Kappa-B P65 Subunit - *RELA*), endothelial function (endothelin1 - *EDN-1*) and oxidative stress response (Superoxide Dismutase 2 - *SOD2*) (Supplementary Table S1 and Supplementary Fig. 2E–F). Fenofibrate treatment did not significantly alter mRNA expression of PPARα or its target genes at 2d post-treatment. However, by day 7, fenofibrate markedly enhanced *PPARα* and *GLUT1* expression while reducing the expression of several of its transcriptional targets, including *EDN*-1 and *SOD2*. These results support that fenofibrate increases PPARα activity, partly by boosting its phosphorylation.

### 2.4 Fenofibrate changed the proteome of irradiated endothelial cells

**Fig. 3:**
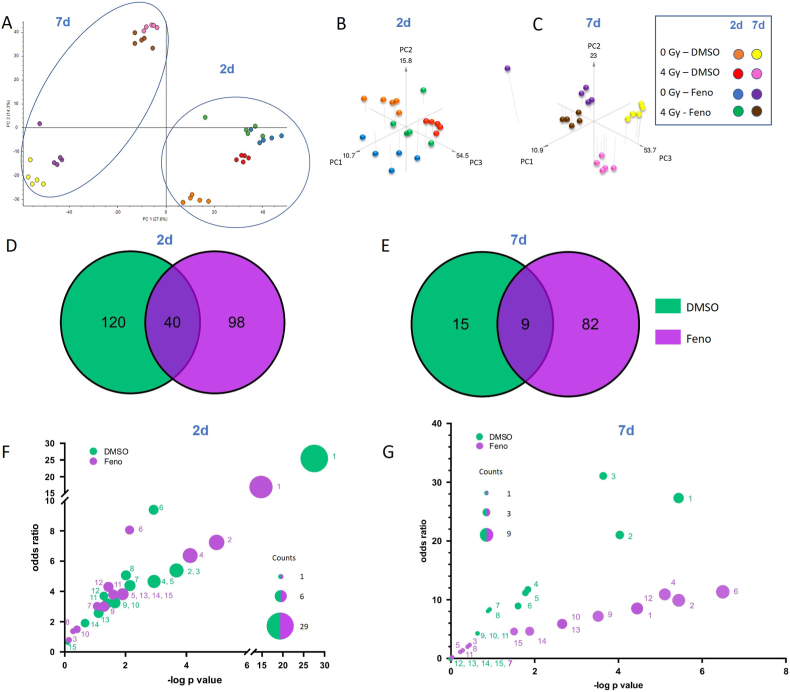
**Changes in the proteome of irradiated HCAECs with and without fenofibrate treatment**. The 2D and 3D PCA were performed on normalized intensities of all proteins. (A–C). The shared proteins between the irradiated DMSO- and 10 μM fenofibrate (Feno) -treated samples were shown in the venn diagram (D–E). The most significant pathways altered in both proteome profiles, 2d (F) and 7d (G) after irradiation were shown. The pathways shown in the plot were ranked by significance in the proteome of irradiated DMSO-treated cells, with additional pathways unique to the fenofibrate-treated proteome included for comparison (Table 1, Table 2). Pathways were compared based on odds ratio and -log p-value, and referenced by numbers in Table 1, Table 2 The size of each bubble represents the number of proteins identified in the corresponding pathway. DMSO shows proteome of 4 Gy irradiated vs sham irradiated DMSO-treated cells. Feno shows proteome of 4 Gy irradiated vs sham irradiated fenofibrate-treated cells. The analyses were generated by Enrichr (https://maayanlab.cloud/Enrichr/).

The proteome of irradiated HCAECs treated with fenofibrate or with the solvent control (DMSO) was analyzed using label-free proteomics. The Principal component analysis (PCA), based on the normalized intensities of all proteins, showed clustering between the different groups (PC1: 27.6 % and PC2: 14.3 %) (Fig. 3A). As marked by circles in Fig. 3A, the proteome profiles were separated in a time-dependent manner. The distinction between the non-irradiated and the 4 Gy irradiated samples was also visible at both time points. To emphasize the effect of fenofibrate on irradiated cells, 3D PCA was also performed for each time point (Fig. 3B and C). The analysis indicated, the fenofibrate-treated samples were distinguished from the DMSO-treated samples. The analysis of HCAECs proteome profiles identified more than 3000 proteins, of which 85% with at least 2 unique peptides. All identified and quantified proteins are shown in Supplementary Table S2. All significantly deregulated proteins at different time-points are shown in Supplementary Table S3–S6.

The proteomics analysis showed that irradiation significantly altered the expression level of 160 (15 up- and 145 down-regulated) and 138 (41 up- and 97 down-regulated) proteins (±1.5-fold; *q* ≤ 0.05) in DMSO- and fenofibrate-treated HCAECs respectively, 2d after exposure to 4 Gy (Supplementary Tables S3 and S4), with 40 deregulated proteins shared amongst the two groups (Fig. 3D). The proteome analysis of irradiated HCAECs after 7d showed that irradiation altered the expression level of 24 (20 up- and 4 down-regulated) and 91 (72 up- and 19 down-regulated) proteins (±1.5-fold; *q* ≤ 0.05) in DMSO- and fenofibrate-treated HCAECs respectively (Supplementary Tables S5 and S6), with 9 deregulated proteins shared amongst the two groups (Fig. 3E).The comparison of the most affected pathways in the proteome of DMSO- and fenofibrate-treated HCAECs 2d after exposure to 4 Gy indicated that fenofibrate altered the significance of enriched signalling pathways such as Myc target genes, glycolysis, mTOR signalling, PI3K-AKT signalling, DNA repair and apoptosis (Fig. 3F and Table 1). Clusters of proteins were enriched in metabolic pathways, including glycolysis, peroxisomes, and oxidative phosphorylation in irradiated cells at 2d post-exposure, which was restored by treatment with fenofibrate. The analysis of affected pathways 7d post exposure to 4 Gy, indicated changes in interferon signalling, Myc target genes, fatty acid metabolism, mTOR-PI3K-AKT signalling pathway and oxidative phosphorylation after treatment with fenofibrate (Fig. 3G and Table 2).

**Table 1:** Pathway enrichment analysis of the proteomes revealed affected pathways in irradiated DMSO- and fenofibrate (Feno)-treated HCAECs 2d after 4 Gy exposure compared with sham-irradiated controls. DMSO shows proteome of 4 Gy irradiated vs sham irradiated DMSO-treated cells. Feno shows proteome of 4 Gy irradiated vs sham irradiated fenofibrate-treated cells.

#	Term	-logpvalue (Feno)	-logpvalue (DMSO)	overlap (Feno)	overlap (DMSO)	odds ratio (Feno)	odds ratio (DMSO)
1	Myc Targets V1	14,64023197	27,59389109	18/200	29/200	16,356912	25,46319361
2	G2-M Checkpoint	5,00299465	3,673638481	9/200	8/200	7,241819372	5,385964912
3	Glycolysis	0,125617731	3,673638481	1/200	8/200	0,726574047	5,385964912
4	E2F Targets	4,141317688	2,93625185	8/200	7/200	6,353682171	4,657421518
5	mTORC1 Signalling	1,910609545	2,93625185	5/200	7/200	3,820512821	4,657421518
6	Myc Targets V2	2,13245426	2,922225058	3/58	4/58	8,062957938	9,395061728
7	DNA Repair	1,075212506	2,143207666	3/150	5/150	3,002741395	4,381535039
8	Pperoxisome	2,91E-01	2,010090143	1/104	4/104	1,410622501	5,061538462
9	Epithelial Mesenchymal Transition	1,310695605	1,647845509	4/200	5/200	3,017799601	3,249793218
10	Oxidative Phosphorylation	0,399195843	1,647845509	2/200	5/200	1,471380471	3,249793218
11	Apoptosis	1,60367595	1,39194459	4/161	4/161	3,774914994	3,214600686
12	PI3K/AKT/mTOR Signalling	1,449197589	1,282592386	3/105	3/105	4,337357331	3,697639565
13	p53 Pathway	1,910609545	1,113150815	5/200	4/200	3,820512821	2,569858713
14	Complement	1,910609545	0,666174233	5/200	3/200	3,820512821	1,905299234
15	Xenobiotic Metabolism	1,910609545	0,096361398	5/200	1/200	3,820512821	0,620745236

**Table 2:** Pathway enrichment analysis of the proteomes revealed affected pathways in irradiated DMSO- and fenofibrate (Feno)-treated HCAECs 7d after 4 Gy exposure compared with sham-irradiated controls. DMSO shows proteome of 4 Gy irradiated vs sham irradiated DMSO-treated cells. Feno shows proteome of 4 Gy irradiated vs sham irradiated fenofibrate-treated cells.

#	Term	-logpvalue (Feno)	-logpvalue (DMSO)	overlap (Feno)	overlap (DMSO)	odds ratio (Feno)	odds ratio (DMSO)
1	Interferon Gamma Response	4,445704951	5,460483074	7/200	5/200	8,512953368	26,69500675
2	Myc Targets V1	5,439832697	4,054813556	8/200	4/200	9,898092369	20,18367347
3	Interferon Alpha Response	0,445917044	3,682209739	1/97	3/97	2,293171296	30,21580547
4	Fatty Acid Metabolism	5,107781786	1,816068655	7/158	2/158	10,90397351	11,55011655
5	Apoptosis	0,282713594	1,80062691	1/161	2/161	1,371458333	11,33047456
6	Oxidative Phosphorylation	6,491359156	1,623990324	9/200	2/200	11,33073682	9,080808081
7	PI3K/AKT/mTOR Signalling	0	0,925397047	0	1/105	0	8,307692308
8	Bile Acid Metabolism	0,397054147	0,899086995	1/112	1/112	1,981781782	7,781041911
9	Glycolysis	3,515202134	0,668712392	6/200	1/200	7,173438448	4,320952589
10	mTORC1 Signalling	2,656305586	0,668712392	5/200	1/200	5,877757901	4,320952589
11	KRAS Signalling Dn	0,221743881	0,668712392	1/200	1/200	1,100502513	4,320952589
12	Adipogenesis	4,445704951	0	7/200	0	8,512953368	0
13	Hypoxia	2,656305586	0	5/200	0	5,877757901	0
14	G2-M Checkpoint	1,87955865	0	4/200	0	4,624208304	0
15	DNA Repair	1,50707949	0	3/150	0	4,583024119	0

### 2.5 Fenofibrate restored the radiation-induced alterations of PI3K-AKT-eNOS signalling

**Fig. 4:**
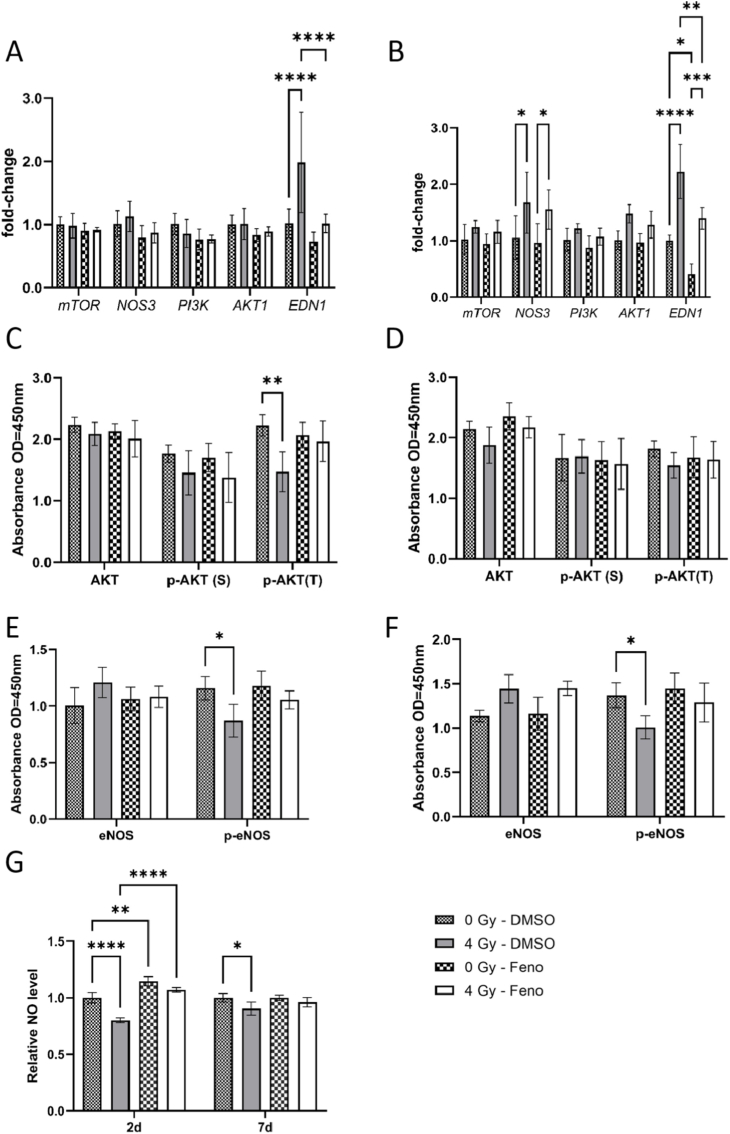
**Analysis of the PI3K-AKT-eNOs pathway.** qPCR and ELISA analysis of the components of PI3K-AKT-eNOS pathway were performed in HCAECs (A–F). The expression level of mRNA was measured using qPCR, 2d (A) and 7d (B) after irradiation with and without fenofibrate (Feno). The expression levels of AKT and p-AKT both serine and threonine and eNOS and p-eNOS proteins were compared using ELISA after 2d (C and E) and 7d (D and F). The levels of NO produced in the HCAECs were measured at the 2d and 7d after irradiation (G). The error bars represent the standard deviation (±SD) (Two-way ANOVA, Tukey's multiple comparisons test; ∗*p* ≤ 0.05; ∗∗*p* ≤ 0.01; ∗∗∗*p* ≤ 0.001; ∗∗∗∗*p* ≤ 0.0001; n = 3).

Proteomic profiling revealed an effect of fenofibrate-treatment on mTOR-PI3K-AKT signalling (Fig. 3F–G and Table 1, Table 2) in irradiated HCAECs at both time points. mTOR-PI3K-Akt contributes to the main function of endothelial cells by regulating endothelial nitric oxide synthase (eNOS) activity and NO production. To validate this finding, we measured the expression of mRNAs and proteins of the pathway, along with the levels of intracellular NO. No changes in the mRNA levels of *mTOR*, *AKT* and *eNOS* were observed after 2d (Fig. 4A), but a significant induction of *eNOS* mRNA was seen in irradiated cells after 7d (Fig. 4B). The effect of radiation on *eNOS* on the mRNA level remained unchanged after fenofibrate treatment (Fig. 4B). In same line, we measured the effect of fenofibrate on the expression of *EDN1*, a potent vasoconstrictive peptide produced by endothelial cells that acts in opposition to NO. Analysis showed, fenofibrate reduced the effect of radiation-induced upregulation of *EDN1* (Fig. 4A and B). Since the proteins of the pathway are induced by phosphorylation, we examined the phosphorylation of AKT (Ser473 and Thr308) and eNOS (Ser1177) in cells using ELISA. The expression level of the phosphorylated (active) form of AKT (p-AKT) (Thr308) and p-eNOS (Ser1177) but not p-AKT (Ser473) were reduced in cells following irradiation. These changes were restored when the cells were treated with fenofibrate (Fig. 4C–F). Irradiation reduced intracellular NO levels at both time points. At 2d, fenofibrate significantly increased NO levels in both irradiated and non-irradiated HCAECs. At 7d, fenofibrate treatment restored the radiation-induced reduction of NO levels (Fig. 4G).

### 2.6 Fenofibrate affects oxidative and nitrosative stress in irradiated cells

**Fig. 5:**
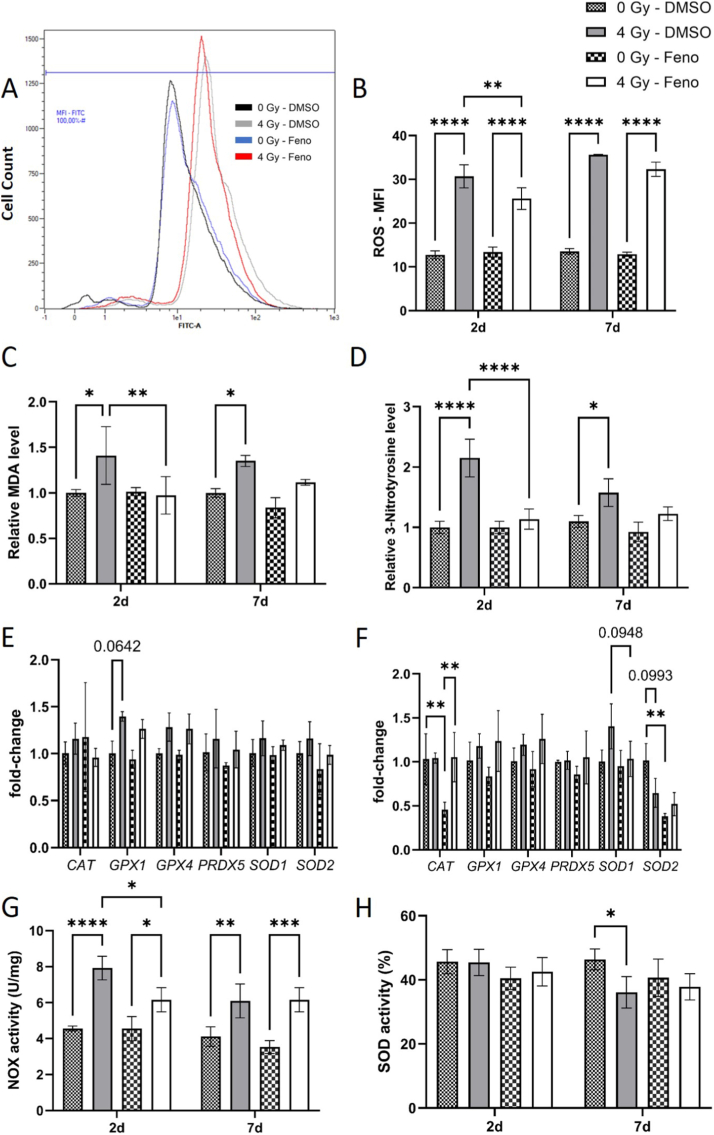
**Analysis of the oxidative stress response.** Levels of intracellular ROS were measured using flow-cytometry in HCAECs, 2d and 7d after irradiation with and without fenofibrate (Feno). An example for the 2d sample set is shown in (A), and quantification for the replicates is shown in (B). Accumulated intracellular oxidative and nitrosative damage were assessed by measuring levels of MDA (C) and levels of 3-NT (D). qPCR analysis of key components of oxidative stress response were performed in HCAECs 2d (E) and 7d (F) after irradiation. Activities of NOX (G) and SOD (H) were measured. The error bars represent the standard deviation (±SD) (Two-way ANOVA, Tukey's multiple comparisons test; ∗*p* ≤ 0.05; ∗∗*p* ≤ 0.01; ∗∗∗*p* ≤ 0.001; ∗∗∗∗*p* ≤ 0.0001; n = 3).

Impairment of NO signalling, commonly occurs alongside elevated oxidative and reactive nitrogen species [22,23]. To validate this finding, the levels of intracellular ROS and MDA as an assessment of accumulated oxidative damage were measured. Our results demonstrated elevated levels of ROS and MDA at both 2d and 7d after irradiation. Fenofibrate treatment significantly reduced ROS and MDA levels at the 2d time point (Fig. 5A–C). Additionally, fenofibrate restored MDA levels to near baseline in cells at the 7d time point (Fig. 5C). We further examined whether enhanced ROS production contributes to NO consumption and whether this effect could be mitigated by fenofibrate. To assess this, we measured 3-nitrotyrosine (3-NT) levels in HCAECs lysates by ELISA as an indirect readout of NO being diverted into reactive nitrogen species. Irradiation significantly increased 3-NT levels at both time points, while fenofibrate treatment markedly reduced them after 2d. A similar trend toward reduction was also observed at 7d, although the difference was not statistically significant. These findings suggest that attenuation of ROS-mediated NO consumption contributes to the restoration of NO availability in our system (Fig. 5D).

To further investigate oxidative stress response mediators, the expression levels of mRNA of some antioxidant enzymes were measured using qPCR (Fig. 5E and F). At 2d, the mRNA levels of Peroxiredoxin-5 (*PRDX5*), glutathione peroxidase (*GPX1*), *GPX4*, *SOD1*, and Catalase (*CAT*) in HCAECs remained unchanged across all groups (Fig. 5D). The fenofibrate treatment reduced the mRNA expression level of *CAT* after 7d in non-irradiated cells (Fig. 5E). To clarify the source of the reduction in total ROS, we performed two additional activity assays, measuring both NADPH oxidase (NOX) and SOD activity in HCAECs. Our results showed that radiation significantly increased NOX activity at both time points, which was well reflected by enhanced levels of ROS and MDA. Treatment with fenofibrate led to a marked reduction in NOX activity in irradiated cells 2d after exposure accompanied by decrease in cellular ROS and MDA (Fig. 5G). In the case of SOD, although fenofibrate reduced SOD2 mRNA levels in sham irradiated cells, no measurable change in total SOD enzymatic activity was observed at day 2, either after irradiation or following fenofibrate treatment. At later time points, irradiation significantly decreased SOD activity in HCAECs, accompanied by elevated MDA and 3-NT levels; these alterations were not significant following fenofibrate treatment (Fig. 5H).

### 2.7 Fenofibrate attenuated activation of interferon pathway in irradiated cells

**Fig. 6:**
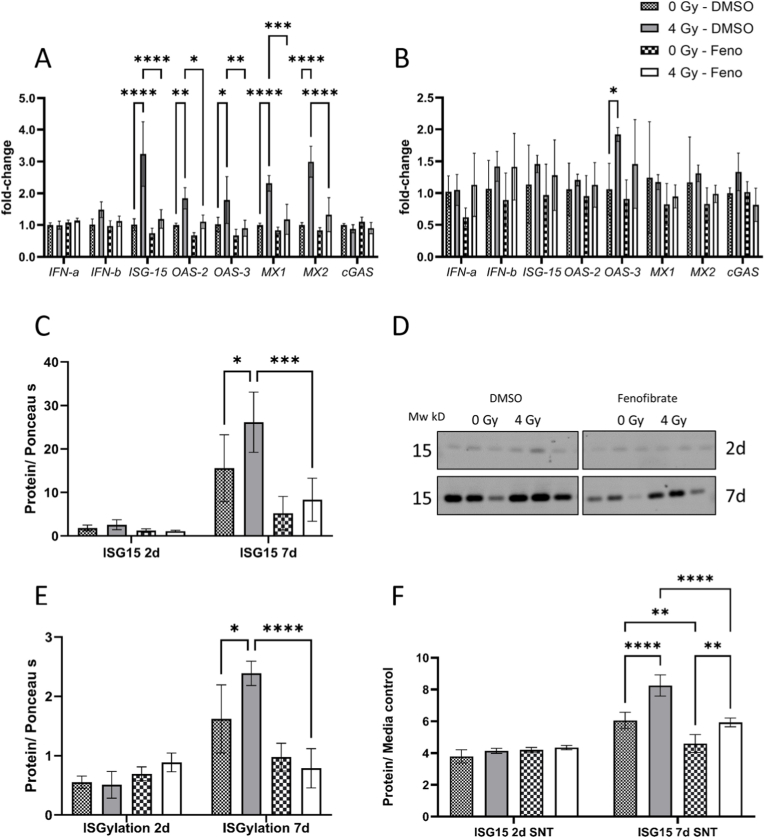
**Analysis of the interferon signalling.** qPCR analysis of the components of interferon pathway were performed in HCAECs, 2d (A) and 7d (B) after irradiation with and without fenofibrate (Feno). The immunoblotting of free ISG15 protein (C and D) and ISGylated proteins 2d and 7d after irradiation (E). The release of ISG 15 in the supernatant (SNT) after 2d and 7d was quantified using a slot-blot (F). The error bars represent the standard deviation (±SD) (Two-way ANOVA, Tukey's multiple comparisons test; ∗*p* ≤ 0.05; ∗∗*p* ≤ 0.01; ∗∗∗*p* ≤ 0.001; ∗∗∗∗*p* ≤ 0.0001; n = 3).

Proteomics data showed differences in the interferon pathway in DMSO- and fenofibrate-treated HCAECs 7d after irradiation (Fig. 3G). To validate the effect of fenofibrate, we analyzed the expression level of mRNA of several components of this pathway including interferon (*IFN*) alpha and beta, Interferon-stimulated gene 15 (*ISG15*), 2′-5′-oligoadenylate synthase 2 (*OAS2*), *OAS3*, Interferon-induced GTP-binding protein Mx1 (*MX1*), *MX2* and Cyclic GMP-AMP synthase (*cGAS*). The analysis showed significant induction of *ISG15*, *OAS2*, *OAS3*, *MX1* and *MX2* 2d after 4 Gy irradiation, fenofibrate attenuated the effect of irradiation on these genes (Fig. 6A). Interestingly the effects of radiation on mRNA level of these candidates were not observed after 7d (except for *OAS3*) suggesting a delayed correlation between mRNA and protein alterations in this pathway (Fig. 6B). We also compared the changes in the levels of free intracellular and released ISG15 protein as well as covalently-bound to target proteins via ISGylation (Fig. 6 C-6E and Supplementary Fig. 3A–3C). The measurement confirmed increased levels of both forms of ISG15 in irradiated cells, 7d after irradiation that is significantly reduced in fenofibrate treated cells (Fig. 6C–E). In good agreement with these findings, ISG15 release was elevated at the 7d time point, but this was significantly reduced in fenofibrate-treated cells compared to DMSO-treated controls (Fig. 6F).

To assess whether the observed effects were specific to the ISG15 pathway or involved other ubiquitin-like modifiers, we analyzed the level of SUMO2/3, SUMO1, and ubiquitination by immunoblotting, examining both free and conjugated forms. Overall, there were no major changes in global SUMOylation, although fenofibrate reduced the levels of free SUMO2/3 at an early time point (2d) and free SUMO1 at a later time point (7d) in irradiated HCAECs (Supplementary Fig. 4A–H). The analysis of ubiquitination revealed an accumulation of polyubiquitinated proteins in irradiated cells at 7d, Fenofibrate treatment markedly reduced this polyubiquitination (Supplementary Fig. 4I–K).

### 2.8 Fenofibrate reduced inflammation in irradiated cells

**Fig. 7:**
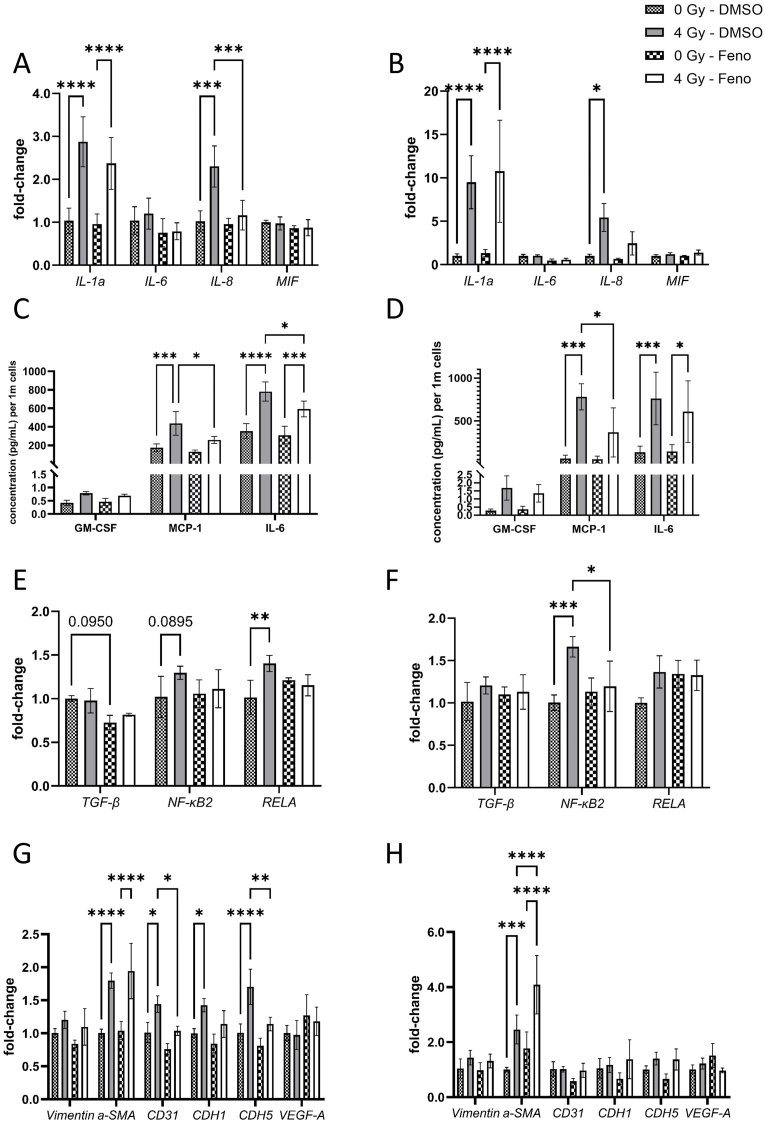
**Analysis of cytokines, interleukins and endothelial mesenchymal transition markers.** qPCR analysis of inflammation markers 2d (A) and 7d (B), inflammatory regulators 2d (E) and 7d (F) and EndMT markers 2d (G) and 7d (H) were performed in irradiated HCAECs with and without fenofibrate (Feno). The release of different cytokines including GM-CSF, MCP-1 and IL-6 which were quantified using flow cytometry after 2d (C) and 7d (D). The error bars represent the standard deviation (±SD) (Two-way ANOVA, Tukey's multiple comparisons test; ∗*p* ≤ 0.05; ∗∗*p* ≤ 0.01; ∗∗∗p ≤ 0.001; ∗∗∗∗*p* ≤ 0.0001; n = 3).

Since the induction of interferons can modulate the activation of interleukins (IL), we compared the expression level of mRNA of *IL1a*, *IL6*, *IL8* and macrophage migration inhibitory factor (*MIF*) in cells. The irradiation induced the *IL1a* at both time points and even after treatment with fenofibrate. In contrast, the induction of *IL8* by irradiation has lost the significance after fenofibrate treatment at both time points. The expression levels of *IL6* and *MIF* remain unchanged (Fig. 7A and B).

Furthermore, to assess the release of inflammatory cytokines into the cell culture supernatant, we measured cytokine levels after irradiation and fenofibrate treatment. Several cytokines and interleukins remained below the detection limit in our assay. However, granulocyte-macrophage colony-stimulating factor (GM-CSF), monocyte chemoattractant protein-1 (MCP-1), and IL-6 were quantifiable. Irradiation led to increased levels of MCP-1 and IL-6 at both time points, whereas fenofibrate treatment attenuated the MCP-1 increase at both time points and reduced IL-6 levels only at 2d. (Fig. 7C and D).

Additionally, the expression levels of regulators for inflammatory response, known to be critical downstream targets of PPARα signalling, including Transforming Growth Factor β (*TGF-β*), nuclear factor kappa B subunit 2 (*NF-κB2*), and NF-κB2 P65 Subunit (*RELA*) were measured. Results showed a significant radiation-induced increase in the expression of *RELA* after 2d, while fenofibrate attenuates this induction (Fig. 7E). For the 7d time-point, only *NF-κB2* showed significant radiation-induced response, which was significantly reduced after fenofibrate treatment (Fig. 7F).

### 2.9 Fenofibrate affected cytoskeletal remodelling involved in EndMT in irradiated cells

Proteomic analysis of endothelial cells 2d after irradiation revealed alterations in proteins typically associated with epithelial–mesenchymal transition (EMT) pathways. As endothelial–mesenchymal transition (EndMT) represents a specialized form of EMT in which endothelial cells acquire mesenchymal characteristics, we considered these changes indicative of EndMT and sought to validate this finding. Here we compared the mRNA expression levels of key EndMT components, including *vimentin*, *α-SMA*, endothelial surface marker *CD31*, epithelial cadherin (*CDH1*), and vascular endothelial cadherin (*CDH5*) as well as Vascular Endothelial Growth Factor A (*VEGF-A*), most potent inducers of this process in our cells. The expression levels of *αSMA*, *CD31*, *CDH1* and *CDH5* were increased in irradiated cells 2d after exposure. While fenofibrate did not affect the changes in *α-SMA*, it reduced the radiation-induced effects on *CD31*, *CDH1* and *CDH5* (Fig. 7G). No changes in mRNA levels of these markers except of *α-SMA* were observed 7d after irradiation (Fig. 7H).

## 3 Discussion

The aim of the study presented here was to examine the effects of fenofibrate, a ligand of PPARα, on radiation-induced vascular damage using an *in-vitro* model of endothelial cells. To the best of our knowledge, this is the first study investigating the effects of fenofibrate treatment on the cardiac endothelial proteome after irradiation.

Endothelial cells, forming the inner lining of blood vessels, are particularly vulnerable to radiation-induced injury, leading to impaired vascular function, inflammation, and tissue damage [24,25]. In this context, pharmacological interventions that maintain endothelial function and suppress inflammation are of particular interest. Synthetic agonists of PPARα, such as fenofibrate, regulate cardiac metabolism and inflammatory haemostasis [11,12]. They have shown therapeutic potential in attenuating atherosclerosis and reducing the coronary complications in high-risk patients [15]. In our study, fenofibrate showed such protective effects at a concentration of 10 μM, lower than the concentration required to achieve anti-atherosclerosis clinical efficacy in humans [14] suggesting a potential for low-dose therapeutic use of fenofibrate to minimize risk of its adverse effects.

PPARα transcriptional activity is known to be modulated by phosphorylation, which can enhance or suppress its function depending on the cellular context and upstream kinase signalling [18,19]. In our study, fenofibrate treatment counteracted the radiation-induced decrease in PPARα phosphorylation at Ser12, suggesting enhancement of its transcriptional activity, as reflected by changes in selected PPARα target genes. This activation was accompanied by improved endothelial cell survival and function, indicating a mechanistic link between PPARα activation and endothelial protection following irradiation. Fenofibrate also modulated apoptosis signalling triggered by radiation, particularly by reducing p53 induction at 2d after exposure and decreasing the number of apoptotic cells observed at 7d after irradiation. The temporal gap between early p53 mRNA changes and the later onset of apoptosis suggests involvement of other apoptotic pathways or reflects a delay between transcriptional regulation and the visible apoptotic response. It is important to note that the effect of fenofibrate was further supported by proteomic data, which revealed fenofibrate-induced alterations in apoptosis-related proteins at both time points. The temporal uncoupling between gene expression and protein expression or activity underscores the importance of post-transcriptional and post-translational regulation, as well as protein stability, in shaping delayed cellular outcomes after radiation. The shifts observed in our data indicate that persistent or late-arising protein-level changes may drive long-term cellular dysfunction despite transient transcriptional signals. The effect of PPARα activation on apoptosis appears to be context-dependent, acting either as an inhibitor or inducer depending on the concentration of the PPARα agonist and the specific cell type or experimental model. In good agreement with our data, PPARα activation has been shown to reduce apoptosis in HUVECs [26], adventitial fibroblasts [27] and in vascular endothelial cell in APOE ^−/−^ mice [28].

Besides the effect of fenofibrate on cell survival, proteomics data indicated changes in pathways involved in endothelial function such NO signalling and EndMT. We observed significant alterations in proteins related to the mTOR-PI3K–AKT pathway, along with a marked reduction in phosphorylation of eNOS and AKT and level of intracellular NO, suggesting disruption of a key signalling axis critical for endothelial function. Treatment with fenofibrate restored AKT phosphorylation exclusively at Thr308, rescued eNOS phosphorylation and increased levels of NO, suggesting that fenofibrate provide a protective effect on the PI3K–AKT–eNOS pathway in irradiated endothelial cells. These findings are significant given the essential role of eNOS-derived NO in vascular homeostasis, including the regulation of vascular tone, inflammation, thrombosis, angiogenesis, and blood pressure [29]. While the ability of PPARα activation to enhance eNOS expression and activity has been demonstrated in other cardiovascular contexts [[30], [31], [32]], our results provide novel evidence that this regulatory mechanism also applies in the setting of radiation-induced endothelial injury. These findings align with previous in vivo observations that fenofibrate can restore radiation-impaired eNOS activity in the mouse heart [17]. Moreover, our data also showed fenofibrate reduced *EDN1* expression in irradiated endothelial cells. EDN1, a potent vasoconstrictor, counteracts the vasodilatory and anti-inflammatory effects of NO [33]. An imbalance characterized by decreased NO and increased EDN1 contributes to endothelial dysfunction and vascular damage [33,34]. Thus, fenofibrate's ability to restore the balance between NO and *EDN1* further supports its protective role in radiation-induced endothelial injury.

Consistent with these effects, we observed significantly increased 3-NT levels in irradiated cells, suggesting NO consumption by ROS [23]. Fenofibrate markedly reduced 3-NT levels at 2d post-irradiation, with a similar but non-significant trend at 7d. Collectively, these findings suggest that radiation decreases NO levels in endothelial cells by disrupting eNOS signaling and enhancing ROS-mediated NO consumption. Fenofibrate effectively counteracted this effect by targeting both mechanisms.

We also showed that fenofibrate interferes cytoskeleton remodelling involved in radiation-induced EndMT, a process implicated in cardiac pathologies such as fibrosis and atherosclerosis. During EndMT, endothelial cells lose markers like CD31 and VE-cadherin, gain mesenchymal markers like α-SMA, and differentiate into fibroblasts or smooth muscle cells [35]. EndMT also affects endothelial integrity and permeability [36]. We observed a simultaneous increase in both *α-SMA* and *CD31* levels in our study 2d after irradiation, suggesting changes in cytoskeleton organization due to cellular stress response. These alterations may trigger EndMT rather than a full transition, which contributes to disease progression [37]. This is supported by the lack of changes in *TGF-β*, a key EndMT driver, in irradiated cells. However, other inflammatory cytokines such as IL-1 and TNF-α, as well as oxidative stress, can also induce EndMT. EndMT is known as a critical link between inflammation and endothelial dysfunction [36] and play a role in the progression of atherosclerosis [38]. The mechanism behind radiation-induced EndMT remains to further analyzed.

Impaired NO signalling, often accompanied by elevated ROS, is a key contributor to vascular pathology [22]. In our study, irradiation increased NOX activity, ROS levels, and accumulated stress damage (MDA and 3NT) at both time points. NOX, a major enzymatic source of ROS in endothelial cells contributes to endothelial dysfunction and vascular diseases [39,40]. Fenofibrate appears to attenuate radiation-induced ROS primarily through suppression of NOX activity. At later time points, radiation-induced alterations in oxidative damage markers (MDA and 3-NT) were no longer significant in fenofibrate-treated cells. This may reflect a secondary enhancement of mitochondrial antioxidant capacity, potentially involving partial recovery of SOD2 activity, even though NOX activity remained elevated. The PPARα activation plays a crucial role in maintaining oxidative stress homeostasis and reducing oxidative damage [41,42]. We have previously shown that fenofibrate reduced oxidative damage in irradiated mouse hearts [17].

Inflammation, especially the interferon signalling pathway, was the main affected pathway following irradiation, particularly at later time points. Our data showed that fenofibrate treatment significantly reduced the radiation-induced inflammatory response in endothelial cells. Fenofibrate mitigated the radiation-induced interferon signalling pathway by targeting its key downstream effector, Interferon-stimulated gene 15 (ISG15). ISG15 exists in multiple forms, including a free (unconjugated) form and a protein-conjugated form known as ISGylation. ISGylation, a post-translational modification analogous to ubiquitination, can alter protein stability and function, while the unconjugated form of ISG15 can act as a pro-inflammatory cytokine, promoting IFNγ secretion and amplifying inflammatory signalling [43,44]. Fenofibrate treatment significantly reduced levels of both intracellular and extracellular free ISG15, as well as ISGylated proteins. Our analysis of other ubiquitin-like modifiers indicates that fenofibrate primarily mediates its restorative effects on the inflammatory response in irradiated cells through modulation of the ISG15 pathway. The transient changes in SUMO proteins and the reduction in polyubiquitination likely reflect additional mechanisms linked to improved protein homeostasis. This supports the conclusion that fenofibrate's protective actions are largely ISG15-dependent, with additional benefits for protein homeostasis.

**Fig. 8:**
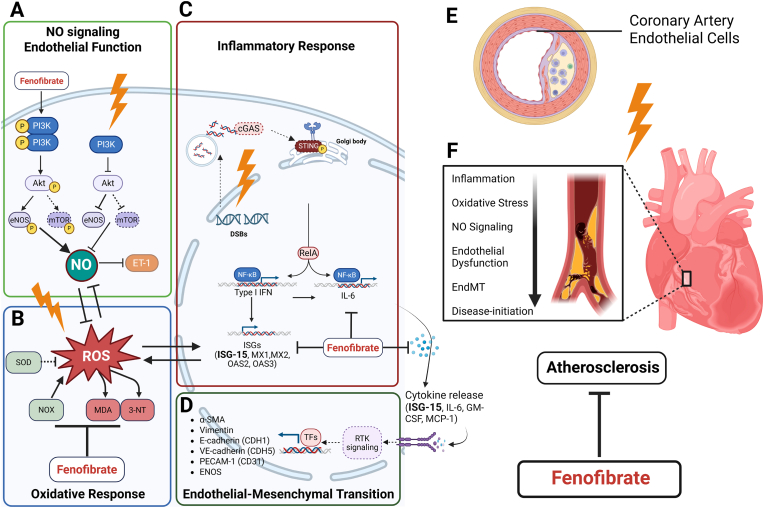
**Proposed mechanism of action of fenofibrate in irradiated HCAECs *in-vitro*.** Irradiation reduced NO signalling via inactivation of the PI3K–AKT–eNOS pathway, whereas fenofibrate reactivated this pathway and restored NO production (A). Consistent with this, irradiation increased ROS generation, NOX activity, MDA levels, and 3-NT levels, while fenofibrate mitigated this effect (B). Irradiation also triggered an inflammatory response, which was counteracted by fenofibrate (C). The released cytokines contribute to inflammation, changes in cytoskeleton organisation and initiation of EndMT, and fenofibrate effectively attenuated the processes (D). Alterations in the pathways described above play a crucial role in the remodelling of vascular endothelial cells involved in the initiation and progression of atherosclerosis. Fenofibrate acts to reduce or restore the effects of irradiation on these pathways (E–F). Solid lines indicate correlations validated in this study, while dashed lines indicate unvalidated correlations.

The ISG15 pathway has been identified as a novel mediator of hypertension-associated vascular damage, contributing to endothelial dysfunction, vascular remodelling, and increased oxidative stress. Mechanistically, it was shown that ISG15 promotes the production of ROS and inflammation, while its modulation affects vascular stiffness, extracellular matrix remodelling, and susceptibility to aortic dilation and aneurysm formation [43,45]. ISG15-deficient mice are protected from angiotensin II–induced hypertension and vascular damage, whereas excessive ISGylation exacerbates fibrosis, inflammation, and aortic rupture. Elevated ISG15 is also observed in human and murine abdominal aortic aneurysms where antioxidant treatment mitigates its detrimental vascular effects, suggesting that ISG15 is a critical for linking interferon signalling, oxidative stress, and hypertensive vascular pathology [43,45]. While the anti-inflammatory effects of PPARα activation are reported in other cardiovascular contexts [46,47], our data highlight here the effect of fenofibrate in irradiated endothelial cells. This also aligns with our previous *in-vivo* findings, where fenofibrate lowered serum levels of inflammatory markers following localized high-dose cardiac irradiation [17]. These findings collectively suggest that administration of PPARα agonist, fenofibrate might have protective effect against endothelial dysfunction, a key pathological event contributing to vascular damage following irradiation. A putative mechanistic model based on the *in-vitro* HCAEC data presented in Fig. 8 showed the interaction between irradiation, fenofibrate and the signalling pathways involved in vascular damage. Irradiation disrupts NO signalling by inhibiting the PI3K-AKT-eNOS pathway, while fenofibrate restores this signalling and enhances NO bioavailability (A). It also reduces radiation-induced ROS production and accumulated oxidative and nitrosative damage (B). Furthermore, fenofibrate suppresses the inflammatory response mediated by ISG-15 and downstream cytokine release (C), which are involved in endothelial-mesenchymal transition (D). As endothelial dysfunction contributes to atherosclerosis development, fenofibrate ability to counteract these effects suggests its therapeutic potential (E and F). Despite these findings, our study has some limitations that should be acknowledged. We only treated the cells with fenofibrate for 1d before irradiation. The effect of fenofibrate with longer treatment or treatment before and after irradiation still needs to be tested. The effect of activation of PPARα must also be investigated in other cardiac cells including cardiomyocytes and fibroblasts and on the interaction between the cells in the irradiated heart.

## 4 Conclusion

Data presented here collectively showed that the activation of PPARα with fenofibrate can reduce the adverse effects of irradiation in endothelial cells. PPARα has demonstrated broad-ranging effects by modulating various radiation-affected pathways, impacting key vascular functions. The results of the present study showed that the radiation-induced alterations in inflammatory and oxidative stress responses, as well as changes in PI3K-AKT-eNOS, EndMT and apoptosis pathways were ameliorated in fenofibrate-treated cells. Taken together, these findings underscore the potential significance of PPARα activation in modulating the vascular response to radiation exposure. Such insights can inform the development of novel strategies aimed at mitigating the adverse effects of radiation on the heart.

## 5 Methods

### 5.1 Cell culture

The HCAECs, primary endothelial cells isolated from the coronary arteries of single donors (Promo Cell, Germany, C-12221) were cultured in ready to use Human Meso Endo Growth Medium (Cell Applications, USA, 212–500) in an incubator with humidified atmosphere at 37 °C with 5 % CO_2_ in concentration of 5000–10000 cells/cm^2^. Cells were monitored regularly and kept in appropriate concentrations with ∼70–80 % maximum confluency levels. For cell passaging, medium was removed and cells were washed with sterile Dulbecco's Phosphate Buffered Saline (DPBS) without Mg^2+^/Ca^2+^ (Biowest, France). TryplE recombinant cell-dissociation enzyme (Thermo Fisher Scientific, USA) was then added and left in the incubator for ∼5 min. Cells were checked for detachment and moved to a falcon tube containing 2x DMEM medium (PAN Biotech, Germany) with 10 % FBS (FBS Premium, PAN Biotech, Germany) to neutralize the enzymatic effects of the TryplE solution. Cells were then centrifuged at 150×*g* for 10 min, the supernatant media was then removed and the cell pellet was resuspended in fresh Human Meso Endo Growth Medium.

### 5.2 Fenofibrate treatment

Fenofibrate (Sigma-Aldrich, F6020-5g) was dissolved in in dimethyl sulfoxide (DMSO) (PAN Biotech, Germany) at a 10 mmol/l stock concentration and stored in aliquots at −20 °C. Working dilutions were freshly prepared in Human Meso Endo Growth Medium on the day of use. For all experiments, fenofibrate-containing media were added 24h after cell seeding, followed by a 24h incubation before irradiation. Control cells received media with equal amounts of DMSO.

### 5.3 Irradiation

Cells were irradiated using the X-ray irradiation cabinet RS225 (Xstrahl Limited, UK). Irradiation was conducted using a 0.5 mm copper filter at 195 kV and 10 mA with the samples positioned 50 cm from the copper filter. For the different experiments, cells were seeded in the desired cell titers and left to incubate for 24h before irradiation. A control sample was included in all experiments at which cells were sham irradiated by being subjected to the same handling and transportation procedures as the irradiated cell samples. The cells were irradiated with a dose rate of 0.5233 Gy/min at room temperature.

### 5.4 Cell count

Cells were counted using Neubauer cell counting chambers. Ten μl of the suspended cells aliquot were mixed with 10 μL of trypan blue, pipetted into the Neubauer counting chamber slide and manually counted. Measurements were taken two times to get the average living cell count.

### 5.5 Cell viability assay

To assess the cellular viability, PrestoBlue High sensitivity (PB-HS) reagent was used (Invitrogen, USA, P50201). HCAECs were seeded at 3000 cells/well in 200 μl media in 96-well plates (4 technical replicates). Cells were treated with fenofibrate and irradiated as described. At 2d and 7d post-irradiation, 10x PrestoBlue High Sensitivity was added to the wells. Plates were protected from light, shaken for 1 min at 300 rpm, incubated for 3h, and measured by a Tecan plate reader (560 nm excitation, 590 nm emission). Media-only wells were treated similarly and used to subtract background.

Additionally, using the same set-up, the cellular viability was measured by CellTiter-Glo® (CTG) Luminescent Cell Viability Assay according to the manufacturer's protocol (Promega, USA, G7570). While PB-HS reagent evaluates cell viability through its reduction by the intracellular reducing environment characteristic of viable cells, the CTG assay quantifies intracellular ATP as a direct measure of metabolic activity.

### 5.6 Cell cycle - Sub G1 analysis

To assess the effects of fenofibrate treatment on the induction of apoptosis, Sub-G1 assay was performed. HCAECs were seeded at 5 × 10^5^ cells in T-75 flasks with 13 ml medium for the 2d time point, and at 4 × 10^5^ cells for the 7d time point. Fenofibrate treatment and irradiation were performed as described above. Afterwards, cells were harvested along with the supernatant in accordance to UCL - London's Global University's protocol. (https://www.ucl.ac.uk/child-health/sites/child_health/files/facilities-flowcyt-subg1_0.pdf). Cell cycle analysis was conducted using MACSQuant Analyzer 10 (Miltenyi Biotec, Germany) and the Sub G1 proportion of the cells was quantified.

### 5.7 Gene expression analysis by real-time qPCR

RNA isolation, reverse transcription polymerase chain reaction (RT PCR), and real-time quantitative polymerase chain reaction (qPCR) were performed as described before [48]. For the synthesis of the first strand cDNA, 1 μg of RNA was used per sample. While for qPCR, the iTaq™ a calculated amount of 1 ng cDNA per reaction was used. mRNA primers were synthesized by Eurofins genomics or purchased from Qiagen (Supplementary Table S1). TATA-box binding protein (*TBP*) was used as a house-keeper gene for internal normalization. Relative gene expression was analyzed using the comparative ΔΔCq. For the normalization, the Cq values of the *TBP* were subtracted from those of the targets of interest.

### 5.8 Fluorescence microscopy

To study the effects of irradiation and fenofibrate treatment on the activity of PPARα, fluorescence microscopy for phospho-PPARα (Ser12) was used. HCAECs were seeded and treated as explained above in 6-well plates with sterile coverslips at the bottom. The cells were washed 2d and 7d after irradiation in the 6 well plates with PBS, fixed in 2 % paraformaldehyde for 15 min at room temperature (RT), washed 3x5 min in PBS+ 0.15 % Triton x-100, and blocked 3x10 min in PBS+1 % BSA+0,15 % Glycine at RT. Cells were then incubated with the primary phospho-PPARα (Ser12) antibody (Invitrogen, USA, PA1-820) in a 1:250 dilution in blocking solution for 3h in RT. Cells were then washed for 5 min in PBS, 10 min in PBS+ 0.15 % Triton X-100, 5 min in PBS, and 5 min in blocking solution. Secondary Alexa Fluor® 488 Conjugate antibody (Cell Signaling Technology, 4412) was added in a 1:1000 dilution and left in the dark for 1h in RT. Cells were then washed 2 × 5 min in PBS+ 0.15 % Triton x-100 and 2 × 10 min in PBS. To stain the actin filaments of the cells, cells were incubated with Phalloidin-iFlour 555 (Abcam, USA, ab176756) for 1h at RT, then washed 3 × 5 min in PBS. Cover slips were then mounted on slides with VECTASHIELD® with DAPI mounting medium.

For visualization and analysis, AxioImager.Z2 microscope was used with the aid of ZEN 2.6 pro software. Images were obtained using the AxioCam 503 mono microscope camera with the same exposure time and sensitivity settings for the samples and later quantified. A minimum of 100 cells were quantified for the 2d time-point and 60 cells for the 7d time-point. To obtain the average fluorescence signal per cell, the total fluorescence signal was quantified and divided by the number of cells per frame.

### 5.9 Immunoblot analysis

Immunoblot analysis was performed as described previously [49] using anti-ISG15 (Thermo Fisher, USA, PA5-31865), PPARα (Invitrogen, USA, PA1-820), SUMO1 (Abcam, USA, ab32058), SUMO2/3 (Abcam, USA, ab3742), and Ubiquitin (Cell Signaling Technology, USA, 3936S). The blots were incubated with the appropriate alkaline phosphatase-conjugated secondary antibody (Santa Cruz Biotechnology, USA) for 2 h at room temperature and developed with 1-stepTM NBT/BCIP substrate solution (ThermoFisher, USA) following standard procedures. Reversible Ponceau-s staining was used as a loading control as the usual loading controls such as GAPDH, or tubulin showed changed levels of expression at least in one condition based on the proteomics data. The corresponding Ponceau-s stained membrane for each antibody detection was provided in supplementary figures. Quantification of digitized images of immunoblot bands from four biological replicates was quantified ImageJ software (v1.50f3) (http://rsbweb.nih.gov/ij/).

### 5.10 Proteome profiling

The cells per treatments were lysed using RIPA buffer (ThermoFisher, USA). Protein concentration was determined by the Bradford assay following the manufacturer's instructions (ThermoFisher, USA). Equal amount of protein lysates was subjected to tryptic digest applying a modified filter aided sample preparation (FASP) procedure as described before [50]. Peptides were collected by centrifugation (10 min at 14 000×*g*), acidified with trifluoroacetic acid (TFA), and stored at −20 °C. Data analysis was performed in data-dependent acquisition (DDA) mode on a HFX mass spectrometer as described in Dreher et al., 2024 [51]. Data analysis was performed using Proteome Discoverer 2.5 as described [51] with the following additions: For the final quantifications, we considered proteins identified with at least two unique peptides (FDR 1 %) in a minimum of 40 % of samples in each group. Proteins showing an irradiated-to-control ratio greater than 1.50 or less than 0.66 (*t*-test; q < 0.05) were defined as significantly differentially expressed. The signalling networks were analyzed using Enrichr software (https://maayanlab.cloud/Enrichr/) [52].

### 5.11 eNOS and phospho-eNOS assay

The expression levels of eNOS and Phospho-eNOS (Ser1177) were measured using Phospho-eNOS (Ser1177) and Total eNOS ELISA Kit (ab279779, Abcam, USA) according to the manufacturer's instructions.

### 5.12 AKT and phospho-AKT assay

The expression levels of the AKT and phosphorylated AKT were measured using AKT PathScan® Total AKT1 Sandwich ELISA Kit (#7170), PathScan® Phospho-AKT (Thr308) Sandwich ELISA Kit (#7252) and PathScan® Phospho-AKT1 (Ser473) Sandwich ELISA Kit (#7160, RayBioTech, USA).

### 5.13 Nitric oxide measurement

The intracellular NO was measured by the Nitric Oxide Assay Kit (Abcam, UK, ab272517) as recommended by the manufacturer. The results were normalized to 100 μg protein input per sample.

### 5.14 Oxidative stress detection assay

The levels of reactive oxygen species (ROS) were measured using CellROX Green Reagent (Thermo Fisher Scientific, USA). HCAECs were harvested and treated according to the manufacturer's protocol. Cells were analyzed using MACSQuant Analyzer 10 (Miltenyi Biotec, Germany).

### 5.15 Lipid peroxidation malondialdehyde (MDA) and 3-nitrotyrosine (3-NT) assay

The levels of lipid peroxidation were measured as an assessment for the accumulated oxidative damage using MDA Assay Kit (BioVision, USA, #K739-100) as recommended by the manufacturer. The levels of 3-NT in cell lysates were measured as a marker for nitrosative stress and oxidative damage (Abcam, USA, ab116691). The results for both assays were normalized to 100 μg protein input per sample.

### 5.16 SOD and NOX activity

To assess the balance between oxidative stress and antioxidant defense, levels of intracellular Superoxide Dismutase (SOD) activity and NADH Oxidase (NOX) activity were measured in cell lysates using SOD Activity Assay Kit (Abcam, USA, ab65354) and NOX Activity Assay Kit (Novus Biologicals, USA, #NBP3-25867) according to the manufacturer's protocol. The results were normalized to 100 μg protein input per sample.

### 5.17 Slot-blot

For quantification of secreted ISG15, a slot blot was performed using the Bio-Dot® SF Apparatus (Bio-Rad, USA) following the manufacturer's instructions. HCAEC supernatant from each treatment was loaded onto the apparatus, and vacuum was applied to immobilize proteins on the membrane, followed by washing with TBS. Membranes were blocked with Intercept® (PBS) Blocking Buffer for 2 h, incubated overnight with ISG15 primary antibody (PA5-31865, Thermo Fisher, USA), then with secondary antibody (Starbright B700 anti-rabbit, Bio-Rad Laboratories GmbH) for 1 h. Imaging and visualization were performed as described for immunoblotting. ISG15 intensity was normalized against background using unconditioned medium. Quantification of digitized images of immunoblot bands from four biological replicates was quantified using ImageJ (v1.50f3) software (http://rsbweb.nih.gov/ij/).

### 5.18 Cytokine ELISA

Extracellular release of inflammatory cytokines (e.g., M-CSF, Granzyme B, IFN-γ, IL-2, IL-4, IL-6, IL-10, IL-17A, IL-21, MCP-1 (CCL2), Perforin and TNF-α) in cell culture supernatants were quantitatively measured based on a fluorescent bead-based system using MACSPlex Cytokine Kits (#130-125-800; Miltenyi Biotec, Germany) according to the manufacturer's protocol. Cytokine concentrations were normalized on the cell counts.

### 5.19 Statistical analysis

GraphPad Prism 9.0 was used for all statistical analyses; a two-way ANOVA test was used to compare two groups. Statistical significance was accepted when p < 0.05. Data are presented in figures as means of three biological replicates ± standard deviation (SD), Asterisks represent different levels of statistical significance (∗*p* ≤ 0.05; ∗∗*p* ≤ 0.01; ∗∗∗*p* ≤ 0.001; ∗∗∗∗*p* ≤ 0.0001).

## CRediT authorship contribution statement

**Omar Elsaeed:** Conceptualization, Formal analysis, Investigation, Methodology, Validation, Writing – original draft, Writing – review & editing. **Christine von Törne:** Methodology. **Prabal Subedi:** Methodology. **Louis Wilhelm:** Methodology, Validation. **Lukas Duchrow:** Formal analysis. **Ali Bashiri Dezfouli:** Methodology. **Gabriele Multhoff:** Supervision, Writing – review & editing. **Simone Moertl:** Conceptualization, Funding acquisition, Supervision, Writing – review & editing. **Omid Azimzadeh:** Conceptualization, Investigation, Methodology, Supervision, Writing – original draft, Writing – review & editing.

## Declaration of competing interest

The authors declare no conflict of interest.

## Data Availability

The raw MS data can be accessed from the STOREDB database (study Id = 1210).
