# Supplementary material for: Fenofibrate attenuates the adverse effects of radiation on endothelial cells through modulation of ROS-NO signalling and inflammation
Source: Redox Biol. 2025 Dec 25;89:103994. doi: 10.1016/j.redox.2025.103994 (PMC12808506; doi:10.1016/j.redox.2025.103994)
Supplement: Multimedia component 2 [file mmc2.docx]

**Supplementary figures**


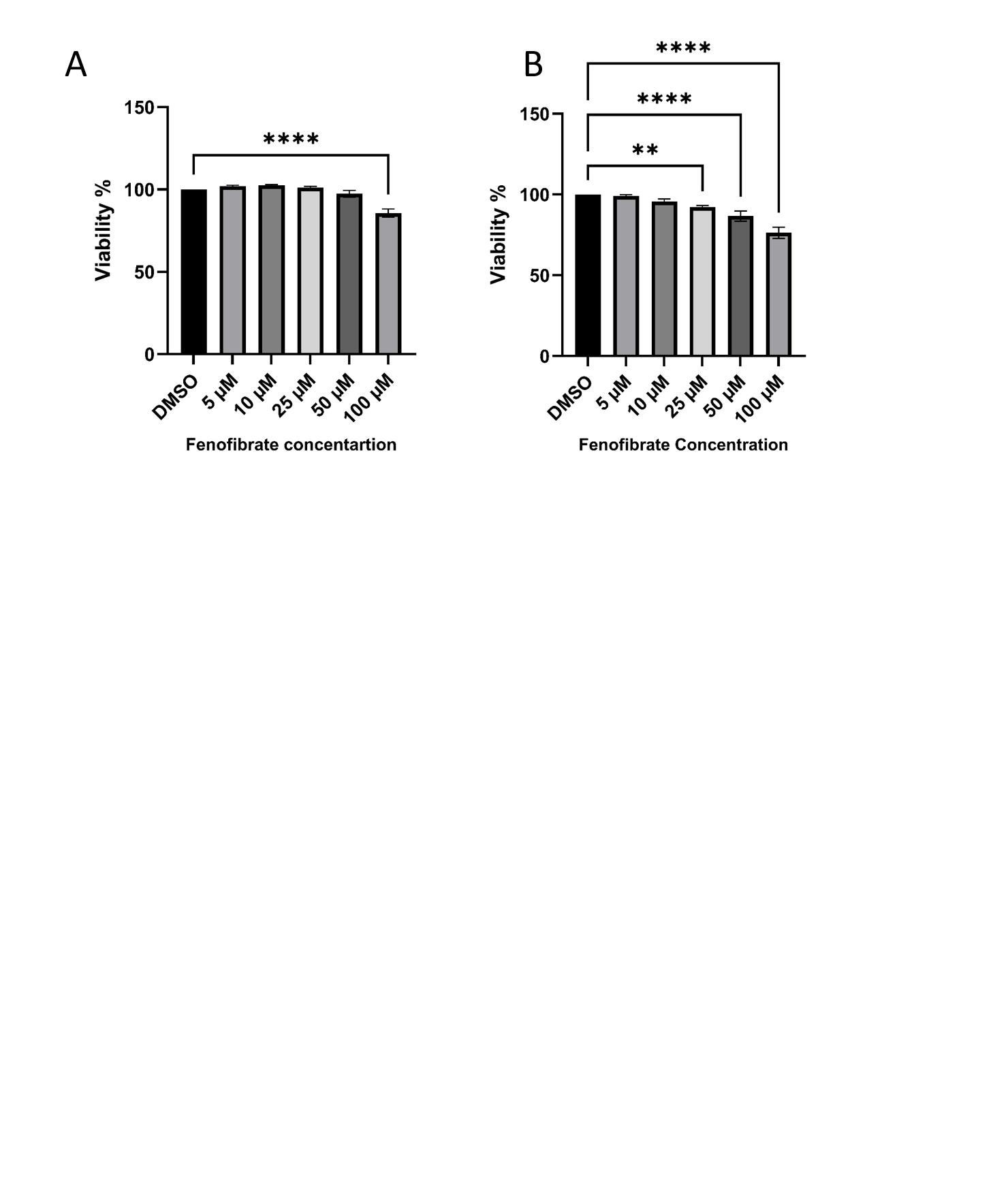


**Supplementary figure 1. The effect of fenofibrate on HCAECs survival.** The survival fraction was compared in HCAECs using Presto Blue viability assay as recommended by the manufacturer 2d (A) and 7d (B) after fenofibrate treatment. The error bars represent the standard deviation (±SD) (Ordinary one-way ANOVA, Dunnett's multiple comparisons test; **p* ≤ 0.05; ***p* ≤ 0.01; ****p*≤ 0.001; *****p* ≤ 0.0001; n=3).


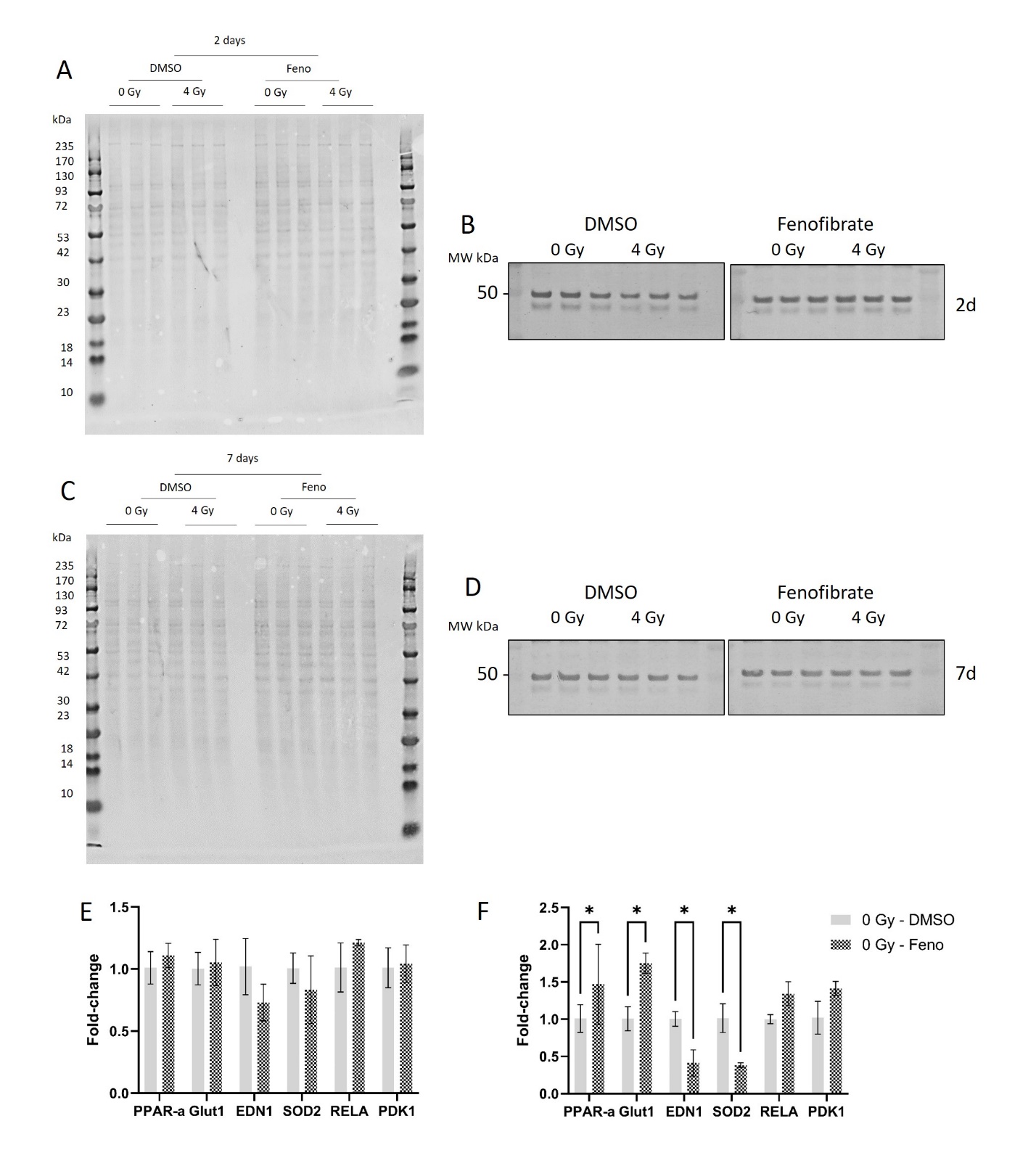


**Supplementary figure 2. The effect of fenofibrate on the** **PPARα phosphorylation and downstream PPARα transcriptional targets**. Levels of free phosphorylated PPARα proteins was compared by immunoblotting in HCAECs (A-D), with total protein measured by Ponceau S staining used as a loading control for 2d (A-B) and 7d (C-D). qPCR analysis of PPARα and PPARα transcriptional targets 2d (E) and 7d (F) after fenofibrate treatment. The error bars represent the standard deviation (±SD) (Unpaired t test, Holm-Šídák multiple correction method; *p ≤ 0.05; n=3).


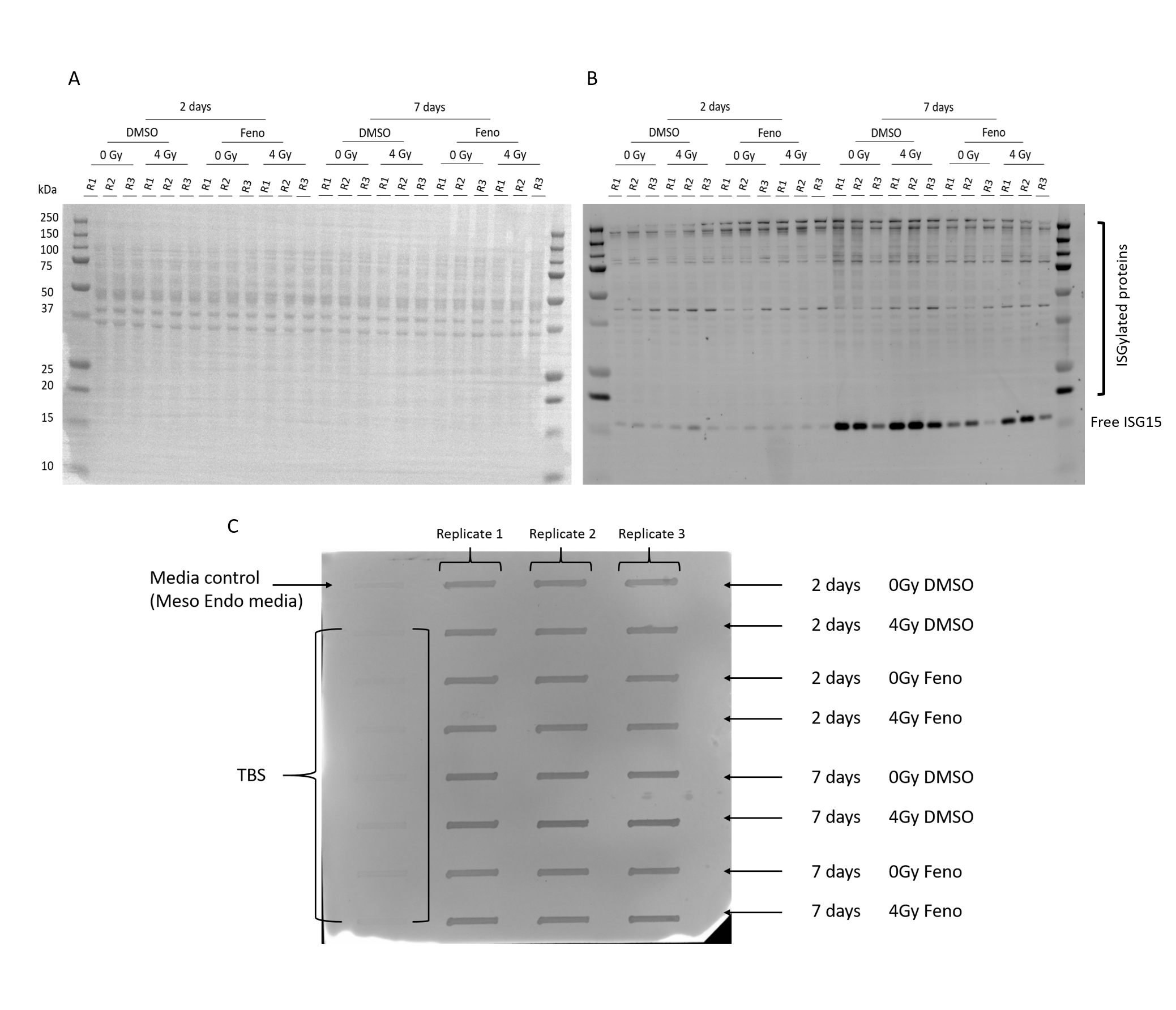


**Supplementary figure 3. The effect of fenofibrate on intracellular ISG15, ISGylated proteins and released ISG15.** Levels of free ISG15 and ISGylated proteins were compared by immunoblotting in HCAECs (A-B), with total protein measured by Ponceau S staining used as a loading control (A). Released ISG15 in the supernatant (SNT) was measured by slot-blot (C), and signal intensity was normalized to background levels determined from unconditioned medium.


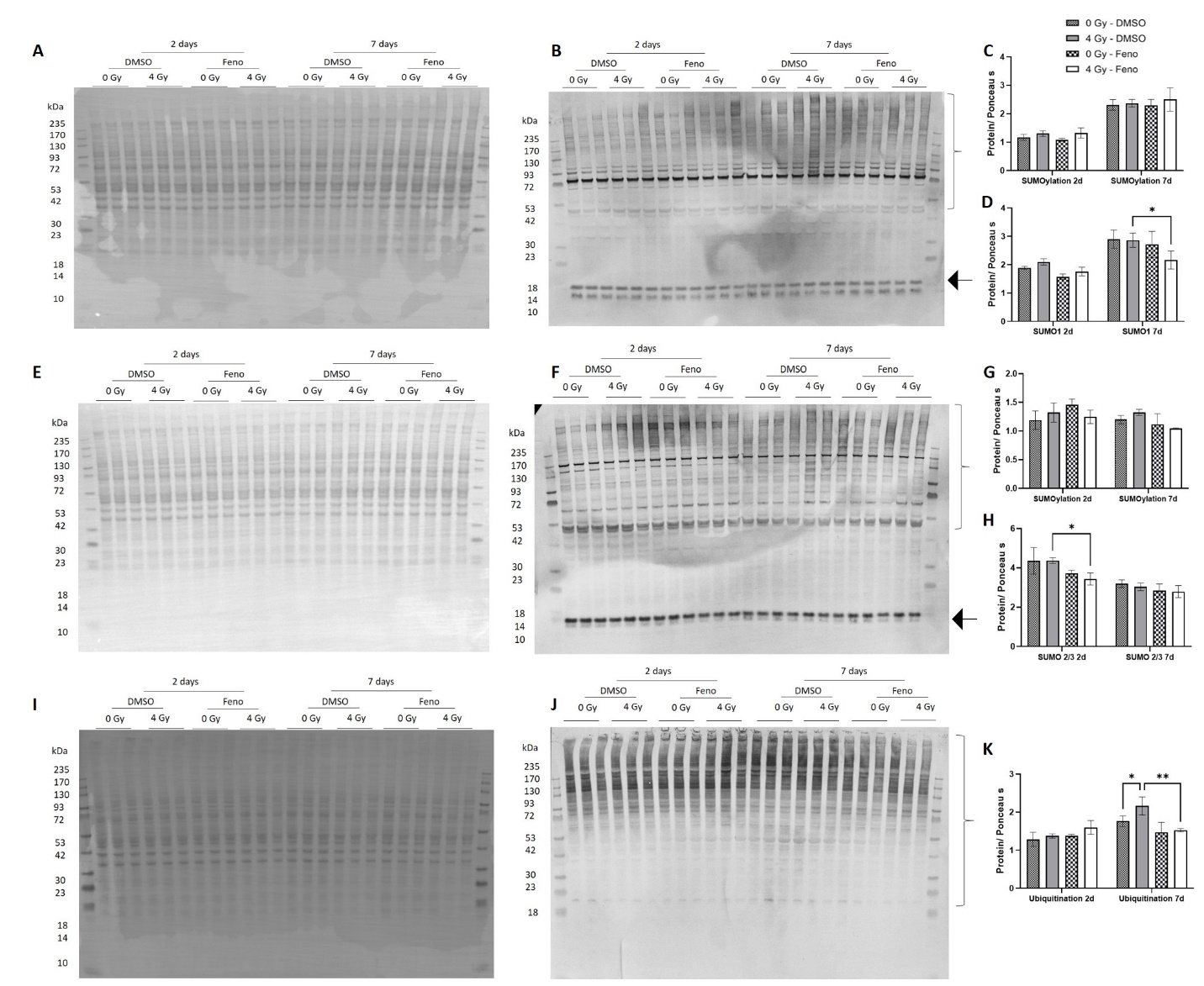


**Supplementary figure 4. The effect of fenofibrate on intracellular SUMO1, SUMO2/3, and ubiquitinated proteins.** Levels of free and conjugated SUMO1 (A–D), SUMO2/3 (E–H), and ubiquitinated proteins (I–K) were analysed in HCAECs by immunoblotting, with total protein visualized by Ponceau S staining used as a loading control (A, E, I). The error bars represent the standard deviation (±SD) (Two-way ANOVA, Tukey's multiple comparisons test; **p* ≤ 0.05; ***p* ≤ 0.01; n=3).
